# Preterm Prelabor Rupture of Membranes and Outcome of Very-Low-Birth-Weight Infants in the German Neonatal Network

**DOI:** 10.1371/journal.pone.0122564

**Published:** 2015-04-09

**Authors:** Kathrin Hanke, Annika Hartz, Maike Manz, Meike Bendiks, Friedhelm Heitmann, Thorsten Orlikowsky, Andreas Müller, Dirk Olbertz, Thomas Kühn, Jens Siegel, Axel von der Wense, Christian Wieg, Angela Kribs, Anja Stein, Julia Pagel, Egbert Herting, Wolfgang Göpel, Christoph Härtel, German Neonatal Network (GNN)

**Affiliations:** 1 Department of Pediatrics, University of Lübeck, Lübeck, Germany; 2 Department of Women’s Health and Obstetrics University of Lübeck, Lübeck, Germany; 3 Department of Pediatrics at University of Kiel, Kiel, Germany; 4 Children’s Hospital Dortmund, Dortmund, Germany; 5 Department of Pediatrics, University of Aachen, Aachen, Germany; 6 Department of Pediatrics, University of Bonn, Bonn, Germany; 7 Children’s Hospital Rostock, Rostock, Germany; 8 Vivantes Children’s Hospital Berlin-Neukölln, Berlin, Germany; 9 Children’s Hospital Auf der Bult Hanover, Hanover, Germany; 10 Children’s Hospital Hamburg-Altona, Hamburg, Germany; 11 Children’s Hospital Aschaffenburg, Aschaffenburg, Germany; 12 Department of Pediatrics, University of Cologne, Cologne, Germany; 13 Department of Pediatrics, University of Essen, Essen, Germany; The Research Institute at Nationwide Children's Hospital, UNITED STATES

## Abstract

**Objective:**

It was the aim of our study to evaluate the independent effect of preterm prelabor rupture of membranes (PPROM) as a cause of preterm delivery on mortality during primary hospital stay and significant morbidities in very-low-birth-weight (VLBW) infants < 32 weeks of gestation.

**Design:**

Observational, epidemiological study design.

**Setting:**

Population-based cohort, German Neonatal Network (GNN).

**Population:**

6102 VLBW infants were enrolled in GNN from 2009-2012, n=4120 fulfilled criteria for primary analysis (< 32 gestational weeks, no pre-eclampsia, HELLP (highly elevated liver enzymes and low platelets syndrome) or placental abruption as cause of preterm birth).

**Methods:**

Multivariable logistic regression analyses included PPROM as potential risk factors for adverse outcomes and well established items such as gestational age in weeks, birth weight, antenatal steroids, center, inborn delivery, multiple birth, gender and being small-for-gestational-age.

**Results:**

PPROM as cause of preterm delivery had no independent effect on the risk of early-onset sepsis, clinical sepsis and blood-culture proven sepsis, while gestational age proved to be the most important contributor to sepsis risk. The diagnosis of PPROM was associated with an increased risk for bronchopulmonary dysplasia (BPD; OR: 1.25, 95% CI: 1.02-1.55, p=0.03) but not with other major outcomes.

**Conclusions:**

The diagnosis of PPROM per se is not associated with adverse outcome in VLBW infants < 32 weeks apart from a moderately increased risk for BPD. Randomized controlled trials with primary neonatal outcomes are needed to determine which subgroup of VLBW infants benefit from expectant or intentional management of PPROM.

## Introduction

Preterm prelabor rupture of membranes (PPROM) occurs in 2–3% of all pregnancies and contributes to 30–40% of preterm births [1]. PPROM is a multifactorial process including certain risk components such as PPROM in previous pregnancy, smoking, socioeconomic status, infection (bacterial vaginosis), amniocentesis, polyhydramnion, multiple gestation and vaginal bleeding. In many cases the cause of PPROM remains unknown. PPROM is initiated by membrane stretch and involves local inflammation and ascending bacterial colonisation [1–2]Women with PPROM between 16 and 26 weeks of gestation were reported to deliver their infant in 79% within four weeks and in 57% within one week after PPROM diagnosis [3]PPROM is associated with a higher risk of prematurity, anhydramnios, cord compression, amniotic infection syndrome (AIS) and placental abruption. PPROM < 20 weeks of gestation causes limb/face deformities, severe lung hypoplasia (Potter´s sequence) and dry lung syndrome which is associated with high mortality and long-term pulmonary complications [4–7].

The initial approach to manage pregnancies complicated by PPROM is based on several parameters assessed upon presentation [8,9]:gestational age, presence of AIS / preterm labor, fetal condition (exposure to antenatal steroids) and availablity of neonatal intensive care. Pregnancies complicated by PPROM ≥ 34 weeks gestational age often receive labor-inducing medication, however, recent data from a randomized controlled trial indicate that this strategy does not substantially improve pregnancy outcomes compared with expectant management [10].Management of PPROM between 22+0 and 34+0 gestational weeks is still a matter of debate. Based on clinical guidelines, PPROM with AIS should receive antibiotics and prompt to termination of pregnancy. PPROM without AIS is closely observed (expectant management) and treated with antibiotics [reviewed in 11].In particular, the treatment for PPROM in the gestational age of 22+0–23+6 weeks is an important aspect of interdisciplinary discussion and needs informed consent of families [8–11].There is currently no evidence on the beneficial use of transabdominal amnioinfusion in women with an (oligo)-hydramnios secondary to PPROM before 26 weeks [10,12].

To what extent PPROM and AIS contribute to adverse outcome is not yet fully understood. AIS and PPROM may be innocent bystanders, as “mild” perinatal inflammation may enhance the functional maturation of the preterm immune system [13]or, vice versa, supposed to be responsible for adverse outcome [14]. A reliable prognosis for VLBW infants delivered after PPROM is difficult to make before birth and in the first hour of life [15]. As up-to-date estimates derived from large-scale population-based studies are scarce, we aimed to determine the role of PPROM as cause of preterm delivery for infant survival and morbidity in very-low-birth-weight (birth weight < 1500g, VLBW) infants < 32 gestational weeks enrolled in the German Neonatal Network (GNN).

## Methods

### VLBW cohort

We performed an observational study and enrolled VLBW infants in a multi-center trial involving 46 neonatal intensive care units in Germany (GNN). The data were collected from infants born between 1^st^ of January 2009 until 31^st^ of December 2012. The inclusion criteria were as follows: birth weight < 1500g and gestational age ≥22+0 and <32+0 weeks, exclusion criteria: lethal malformations, e.g. trisomy 13 and trisomy 18, and maternal causes of preterm delivery, e.g. placental abruption, HELLP (highly elevated liver enzymes and low platelets) syndrome and pre-eclampsia. After written informed consent was given by the parents, infants were enrolled in GNN by the attending physicians and a predefined clinical data set of 220 parameters including antenatal/postnatal treatment and outcome data were recorded by according data sheets. After discharge, data sheets were sent to the GNN center in Lübeck. A basic data set of other infants born in GNN centers but not enrolled in GNN including birth weight, gestational age, and major outcomes including cause of death was documented. A physician trained in neonatology evaluated the data quality by annual on site monitoring of data sets.

### Definitions

Preterm prelabor rupture of membranes (PPROM) was defined as non-iatrogenic rupture of membranes before the onset of labor effective contractions. Gestational age was calculated from the best obstetric estimate based on early prenatal ultrasound and obstetric examination. Small-for-gestational age (SGA) was defined as a birth weight less than 10^th^ percentile for gestational age according to gender-specific standards for birth weight by gestational age in Germany [16]. Amniotic infection syndrome (AIS) was defined as maternal fever (≥ 38.0°C), increased maternal inflammatory markers without any other cause (CRP > 10 mg/L or elevation of white blood cell count > 16000 / μL), fetal or maternal tachycardia, painful uterus and foul smelling liquor. Clinical sepsis was defined as condition with at least two signs of systemic inflammatory response (temperature > 38°C or < 36.5°C, tachycardia > 200/min, new onset or increased frequency of bradycardias or apneas, hyperglycemia > 140 mg/dl, base excess < -10 mval/l, changed skin color, increased oxygen requirements) and neonatologist´s decision to treat with antibiotics for at least 5 days but no proof of causative agent in blood culture and one laboratory sign [17,18]. Blood culture confirmed sepsis was defined as clinical sepsis with proof of causative agent in the blood culture. If coagulase-negative staphylococci (CoNS) were isolated as single pathogen in one peripheral blood culture, two clinical signs and one laboratory sign (platelet count < 100/nl, C-reactive protein > 20 mg/L, immature/total neutrophil ratio > 0.2, white blood cell count < 5/nl) were required for classification of CoNS sepsis.

Early-onset sepsis (EOS) was defined as blood-culture confirmed sepsis occurring within the first 72 h of life. Late-onset sepsis (LOS) was defined as blood-culture confirmed sepsis occurring ≥72 h of life.

Death was defined as all-cause mortality occurring after admission to NICU before discharge home. BPD was diagnosed when needing oxygen at 36 weeks of post menstrual age [19]. Intracerebral haemorrhage grades I-IV were diagnosed according to the ultrasound criteria of Papile [20]. Periventricular leukomalacia (PVL) was diagnosed by means of head ultrasound and/or MRI and included specific findings such as echodense intraparenchymal lesions, periventricular leukomalacia and porencephalic cysts with or without intraventricular hemorrhage.

Antenatal steroids was defined as maternal treatment with betamethasone or dexamethasone for lung maturity of the fetus irrespective of number of doses. Antenatal antibiotics and tocolytics were defined as maternal treatment with antibiotics or tocolytics within the time frame of ≤ 24h before delivery.

### Statistical analysis

Data analysis was performed using the SPSS 20.0 data analysis package (Munich, Germany). Differences between infants born after PPROM and infants without PPROM were evaluated with Chi-square test, Fisher´s exact test and Mann-Whitney U test. A *p* value < 0.05 was considered as statistically significant for single tests. To determine the independent impact of PPROM on outcomes, we performed multivariable logistic regression analyses including well-established risk factors of adverse short-term outcomes in the whole cohort of infants < 32+0 gestational weeks enrolled in GNN.

### Ethics

Written informed consent was obtained from parents on behalf of the infants enrolled in our study. The study parts were approved by the local committee on research in human subjects of the University of Lübeck (08–022; 03.12.2010) and the local ethical committees at the other study centers.

Specifically: Ethical Board of the Medical Chamber of the North Rhine region, Ethical Board of the University of Aachen, Ethical Board of the University of Bonn, Ethical Board of the Medical Chamber of the federal state of Mecklenburg-Vorpommern, Ethical Board of the Medical Chamber of Berlin, Ethical Board of the University of Magdeburg, Ethical Board of the University of Halle, Ethical Board of the University of Tübingen, Ethical Board of the Medical School Hannover, Ethical Board of the University of Cologne, Ethical Board of the University of Essen, Ethical Board of the Medical Chamber of the Westphalia-Lippe region, Ethical Board of the Medical Chamber of Hamburg, Ethical Board of the Medical Chamber of the federal state of Hessen, Ethical Board of the Medical Chamber of the federal state of Baden-Württemberg, Ethical Board of the Medical Chamber of the federal state of Bavaria, Ethical Board of the Saar University.

## Results

### Clinical characteristics

During the observational study period from January 2009 until December 2012 6102 VLBW infants were enrolled in GNN. 4120 infants fulfilled criteria for primary analysis (< 32 gestational weeks without maternal causes of preterm delivery). As shown in Table 1, infants without PPROM (n = 2559) were more likely to be multiples and SGA while infants born after PPROM (n = 1561) were younger at birth.

**Table 1:** Clinical characteristics of VLBW cohort categorized according to PPROM.

Clinical characteristics	No PPROM	PPROM	p-value
No. of infants	2559	1561	
Gestational age (weeks), mean (SD)	28.1 (2.3)	27.7 (2.3)	0.01*
Birth weight (g), mean (SD)	1005 (301)	1045 (295)	< 0.001*
SGA (< 10th percentile, %)	15.6	4.7	< 0.001
Male gender (%)	51.7	54.3	0.11
Multiple birth (%)	41.2	35.1	<0.001
Inborn (%)	97.3	98.3	0.04
Antenatal steroids (%)	91.2	92.9	0.05
Spontaneous delivery (%)	11.0	12.4	0.22
Elective Caesarean Section (%)	79.6	79.3	0.9
Emergency Caesarean Section (%)	9.3	8.2	0.2
Umbilical artery pH (median, IQR)	7.33 (7.28–7.37)	7.34 (7.30–7.38)	0.10*
Maternal background, German (%)	75.2	72.8	0.08

p-values are derived from Fisher´s exact test or Mann-Whitney-U-test if indicated (*)

### Treatment of infants with PPROM

As demonstrated in Table 2, infants with PPROM had a higher likelihood to be exposed to tocolytics and antenatal antibiotics in the time frame ≤ 24h before delivery. The same holds true for more frequent treatment with postnatal antibiotics and inotropes in the “PPROM-group”. These differences may be a consequence of the increased rate of AIS in the infants born after PPROM. There were no differences with regard to other treatment strategies including transfusions, steroids, diuretics or surfactant.

**Table 2:** Treatment differences of VLBW cohort categorized according to PPROM.

Clinical characteristics	No PPROM	PPROM	p-value
No. of infants	2542	1545	
Tocolytic treatment of mother (%)	51.8	68.1	<0.001
Labour refractory to tocolytics(%)	40.8	46.8	<0.001
Amniotic infection syndrome (%)	17.0	50.1	<0.001
Antenatal antibiotics (%)	46.2	81.0	<0.001
Postnatal antibiotics (%)	88.7	95.9	<0.001
Inotrope support (%)	20.2	23.1	0.03
Minimal MAP first 24h, median (IQR	27 (23–31)	26 (22–31)	0.31*
Diuretics (%)	36.8	36.6	0.90
Transfusions (%)	48.1	48.7	0.89
No. of transfusions, median (IQR)	0 (0–2)	0 (0–2)	0.82
Hydrocortisone (%)	10.7	11.6	0.41
Dexamethasone (%)	5.3	6.0	0.41
Prednisolone (%)	3.2	3.4	0.82
Surfactant received (%)	65.2	65.2	0.91
surfactant applications, median (IQR)	1 (1–2)	1 (1–2)	0.92*
Endotracheal ventilation (%)	53.4	55.4	0.20
Duration ventilation, median (IQR) d	1 (0–7)	2 (0–8)	0.40*
Duration oxygen supplement	17 (3–53)	22 (3–55)	0.21*

p-values are derived from Fisher´s exact test or Mann-Whitney-U-test if indicated (*)

### Short-term outcomes in infants with PPROM

No differences were noted with regard to short term outcomes in both groups stratified to PPROM (Table 3). In Table 4 we describe an analysis restricted to infants born after PPROM. Infants born after PPROM with concurrent AIS had a lower gestational age and birth weight compared to those infants born after PPROM without AIS. There were no differences in outcomes apart from a higher risk for clinical sepsis in AIS infants, particularly in the first 72h of life (early onset clinical sepsis, 18.2 vs. 12.9%, p = 0.004), and a longer need for O_2_ supplement.

**Table 3:** Short-term outcomes categorized according to PPROM.

Clinical characteristics	No PPROM	PPROM	p-value
No. of infants	2526	1542	
Intracerebral haemorrhage (%)	18.5	19.1	0.64
ICH Grade I (%)	7.0	7.3	
ICH Grade II (%)	4.6	4.6	
ICH Grade III (%)	3.1	3.4	
ICH Grade IV (%)	3.6	3.5	
PVL (%)	3.1	3.2	0.80
Early-onset sepsis (%)	1.9	2.6	0.11
Pneumonia < 72 h (%)	0.8	0.4	0.5
Late-onset Pneumonia (%)	3.5	4.3	0.12
Clinical sepsis (%)	34.0	36.1	0.18
Blood-culture proven sepsis (%)	13.4	14.2	0.46
Late-onset sepsis (%)	12.6	13.0	0.75
Pneumothorax (%)	5.4	6.5	0.15
O_2_ supplement, median (IQR) days	15 (3–50)	20 (2–51)	0.23*
BPD (%)	13.6	15.0	0.94
Death (%)	4.1	4.9	0.21

p-values are derived from Fisher´s exact test or Mann-Whitney-U-test if indicated (*)

**Table 4:** Short-term outcome in infants born after PPROM categorized according to diagnosis of amniotic infection syndrome (AIS).

Clinical characteristics	no AIS	AIS	p-value
No. of infants	777	781	
Gestational age (weeks), mean (SD)	28.1 (2.3)	27.4 (2.3)	< 0.001*
Birth weight (g), mean (SD)	1074 (300)	1017 (294)	< 0.001*
Intracerebral haemorrhage (%)	18.8	19.3	0.80
ICH Grade I (%)	7.1	7.4	
ICH Grade II (%)	4.7	4.6	
ICH Grade III (%)	3.8	3.1	
ICH Grade IV (%)	2.8	4.1	
PVL (%)	2.6	3.7	0.25
Early-onset sepsis (%)	2.2	3.1	0.34
Pneumonia < 72 h (%)	0.4	0.4	0.9
Late-onset Pneumonia	3.8	4.9	
Clinical sepsis (%)	33.5	38.7	0.035
Blood-culture proven sepsis (%)	13.4	14.8	0.47
Late-onset sepsis (%)	12.6	13.4	0.71
Pneumothorax (%)	6.6	6.4	0.84
O_2_ supplement, median (IQR) days	17 (3–50)	27 (3–60)	0.012*
BPD (%)	14.9	15.0	0.99
Death (%)	4.9	5.0	0.99

p-values are derived from Fisher´s exact test or Mann-Whitney-U-test if indicated (*)

To determine whether there is an independent effect of PPROM on the risk of adverse outcomes we tested PPROM along with well-known influencing factors (gestational age in weeks, birth weight, antenatal steroids, inborn delivery, multiple birth, center, gender and SGA). In our analysis, PPROM had no independent effect on the risk of blood-culture confirmed total sepsis incidence and EOS. In addition to that, PPROM had no influence on total clinical sepsis incidence and clinical sepsis < 72 h (555 cases /3350 controls; OR 1.11, 95% CI: 0.92–1.35, p = 0.28). Gestational age proved to be the most important contributor to sepsis risk (Table 5).

**Table 5:** Logistic regression analysis for sepsis-related outcomes including well known-risk factors.

Outcomes	Early onset Sepsis	Clinical Sepsis	Blood culture proven Sepsis
No. affected infants / controls	80/3823	1368/2554	533/3370
PPROM	OR 1.22 (0.77–1.93) p = 0.39	OR 1.07 (0.92–1.24) p = 0.41	OR 0.93 (0.76–1.13) p = 0.46
Gestational age (per week)	OR 0.74 (0.60–0.90) p = 0.003	OR 0.81 (0.76–0.86) p<0.001	OR 0.80 (0.73–0.86) p<0.001
Birth weight	OR 1.00 (0.99–1.00) p = 0.66	OR 0.99 (0.99–0.99) p<0.001	OR 1.00 (0.99–1.00) p = 0.19
Antenatal steroids	OR 0.51 (0.27–0.97) p = 0.04	OR 0.63 (0.48–0.82) p = 0.001	OR 0.90 (0.65–1.26) p = 0.54
Multiple birth	OR 0.57 (0.34–0.97) p = 0.04	OR 0.85 (0.73–0.99) p = 0.03	OR 0.96 (0.79–1.17) p = 0.68
Inborn	OR 1.34 (0.30–5.90) p = 0.70	OR 0.79 (0.49–1.27) p = 0.33	OR 0.67 (0.38–1.18) p = 0.17
Center	OR 1.03 (1.00–1.05) p = 0.006	OR 1.01 (0.99–1.01) p = 0.10	OR 1.00 (0.99–1.01) p = 0.77
Gender	OR 1.13 (0.72–1.78) p = 0.61	OR 0.83 (0.72–0.96) p = 0.01	OR 1.06 (0.88–1.29) p = 0.54
SGA	OR 1.28 (0.54–3.08) p = 0.58	OR 1.12 (0.84–1.50) p = 0.45	OR 0.98 (0.67–1.41) p = 0.88

As outlined in Table 6, we analysed the independent effect of PPROM on other major outcomes of VLBW infants adjusted to significant clinical confounders in multivariable prediction models. The diagnosis of PPROM was associated with an increased risk for BPD. This effect is not seen in the much smaller subgroup of infants < 27 weeks (388 BPD cases/ 910 no BPD infants; OR: 1.14, 95% CI: 0.87–1.49, p = 0.35), while PPROM is predictive for BPD in infants born 27+0–31+6 weeks of gestation (163 BPD cases / 2364 no BPD infants; OR: 1.90, 95% CI:1.29–2.79, p = 0.001)

**Table 6:** Multivariable logistic regression analysis for mortality and morbidities including well known-risk factors.

Outcomes	Mortality	IVH	PVL	BPD
No. affected infants /controls	173/3745	729/3187	133/3774	561/ 3554
PPROM	OR 1.31 (0.94–1.83) p = 0.12	OR 0.93 (0.78–1.11) p = 0.42	OR 0.80 (0.55–1.16) p = 0.25	OR 1.25 (1.02–1.55) p = 0.03
Gestational age (per week)	OR 0.82 (0.71–0.94) p = 0.004	OR 0.70 (0.65–0.75) p<0.001	OR 0.79 (0.68–0.92) p = 0.003	OR 0.89 (0.82–0.97) p = 0.006
Birth weight (100g steps)	OR 0.99 (0.99–0.99) p<0.001	OR 1.00 (1.00–1.01) p = 0.46	OR 1.00 (0.99–1.00) p = 0.44	OR 0.99 (0.99–0.99) p<0.001
Antenatal steroids	OR 0.93 (0.54–1.59) p = 0.79	OR 0.54 (0.41–0.72) p<0.001	OR 0.59 (0.35–1.00) p = 0.05	OR 1.07 (0.75–1.54) p = 0.70
Multiple birth	OR 1.63 (1.17–2.27) p = 0.003	OR 1.33 (1.11–1.58) p = 0.02	OR 0.94 (0.65–1.36) p = 0.75	OR 0.96 (0.78–1.18) p = 0.69
Inborn	OR 0.53 (0.22–1.29) p = 0.16	OR 0.94 (0.55–1.60) p = 0.83	OR 1.04 (0.36–3.06) p = 0.94	OR 0.55 (0.30–1.02) p = 0.06
Center	OR 0.99 (0.98–1.01) p = 0.24	OR 1.00 (1.00–1.01) p = 0.05	OR 1.02 (1.00–1.04) p = 0.007	OR 1.02 (1.00–1.03) p<0.001
Female	OR 0.64 (0.46–0.90) p = 0.009	OR 0.82 (0.69–0.98) p = 0.03	OR 0.99 (0.69–1.41) p = 0.95	OR 0.55 (0.45–0.68) p<0.001
SGA	OR 1.40 (0.81–2.42) p = 0.23	OR 0.75 (0.53–1.08) p = 0.12	OR 1.13 (0.56–2.29) p = 0.74	OR 1.13 (0.79–1.61) p = 0.52

PPROM had no significant impact on the risk of IVH, PVL and mortality. Major contributors to mortality risk were gestational age, birth weight, gender and multiple birth, while the risk for IVH was associated with multiple birth, gestational age, gender and treatment with antenatal steroids.

## Discussion

### Main findings

Our study presents observational data of the large-scale multicenter GNN cohort evaluating the effect of PPROM as cause of preterm delivery on morbidity and mortality in very-low-birth-weight infants < 32 weeks. Our data suggest that PPROM per se is not associated with adverse outcome. We determined a moderately increased risk for development of BPD associated with PPROM which is restricted to infants born 27+0–31+6 weeks.

### Strengths and limitations

The major strengths of our observation are the large cohort size of a multicenter setting and the accurate phenotypic characterization of infants. This is achieved by regular on-site monitoring of data quality. PPROM management in the most vulnerable gestational age groups is still a matter of debate as conclusive studies are lacking [21–23]. Population-based registry data as presented here cannot overcome this problem but provide important epidemiological information to design a future randomized controlled trial. Thus limitations of our approach need to be thoroughly discussed. In our study, PPROM was defined as non-iatrogenic rupture of membranes before the onset of labor effective contractions. The duration and the cause of PPROM as well as the stratification into anhydramnion or oligohydramnion were not recorded. Therefore the decision to treat PPROM expectantly or intention to treat expectantly cannot be extracted from our main data set. The study findings imply that PPROM itself has no additional impact on adverse outcome adjusted to gestational age and other known risk factors. Previous retrospective single centre cohort studies revealed conflicting results. Some investigators found increased infectious morbidity and mortality in PPROM patients [24,25] while another study noted no adverse outcome in PPROM patients as compared to infants without PPROM [26]. In line with previous observations, gestational age proved to be the most important risk factor for NICU mortality and major neonatal morbidity [27]. The effect of PPROM on BPD risk—independent of gestational age, AIS, antenatal steroids, gender and other contributors—is a major finding of our study, but is only restricted to the smaller subgroup of infants born between 27 and 32 weeks of gestation. This aspect has had a long-standing history of discussion and mainly reflects the presumed association between PPROM, chorioamnionitis and BPD [14]. Notably, a meta-analysis [28]noted significant publication bias and therefore concluded that despite a large body of evidence, “chorioamnionitis cannot be definitively considered a risk factor for BPD”, and the same holds true for PPROM in our perspective.

Currently, the main clinical question whether pregnancies that are aggressively managed differ in infant outcomes than those managed more conservatively still remains unanswered [21, 22]). Ideally, this comparison would take place in the context of a randomized trial. In an observational study, the comparison should be between patients managed expectantly and PPROM patients that might have been managed expectantly, but were not. Our “PPROM” cohort presumably included a mix of patients with a large proportion of infants with surrogate parameters of expectant management (treatment with antenatal steroids: 92.9%, being inborn: 98.3%; elective Caesarean section: 79.3%, no further administration of antenatal antibiotics until < 24h before delivery: 19%) The best observational design would have been to compare outcomes between PPROM managed following different protocols with different thresholds for obstetrical interventions. Our neonatal network lacks these monitored obstetrical data as neonatal but not maternal case record files were the primary data source. For future studies we amended the documentation sheets which will allow to compare outcomes for newborns with different durations of PPROM and thus arguably provide some insight into how to manage PPROM.

Another limitation of our study is potential selection bias which is regarded as major challenge in population-based data. For example, NICU mortality in our study cohort is low and does not fully reflect the mortality rate of infants born in GNN centers. Specifically, the study enrolled 69.5% of infants born in centers of GNN (2355/7702, <32 weeks, 2009–2012). A basic data set including major outcomes (but not cause of preterm delivery) was documented for non-enrolled patients. Notably, the mortality during primary stay in hospital differed remarkably between enrolled patients (4.2%) and non-enrolled infants (25.6%). This is mainly explained by a large proportion of early death in non-enrolled patients due to primary palliative (comfort) care (18.6% of deaths), congenital anomalies (6.2% of deaths) and RDS/early respiratory failure which accounted for 24.6% of NICU deaths. Other less acute causes of death in non-enrolled patients included pulmonary haemorrhage (5.3%), sepsis (15%), NEC/FIP (8.5%), higher grade IVH (10.2%), BPD (2.2%), and others (8.9%).

Another important aspect for future design of randomized trials is the correct diagnosis of AIS following PPROM. Prolongation of pregnancy (to avoid morbidity associated with prematurity) in line with prevention of foetal infection is a major challenge in obstetric management of PPROM. Clinicians are thereby faced with a crucial diagnostic problem of relevant AIS/chorioamnionitis/fetal inflammation, Our analysis revealed that 50% of infants born after PPROM were also exposed to AIS. However, only 2.6% developed EOS while 14% had a clinical sepsis within the first 72 hours of life. Infants with PPROM with or without concurrent AIS did not differ remarkably in outcomes in our setting. Furthermore, AIS was not found to be an independent risk factor for major neonatal complications (data not shown) indicating that current diagnostic criteria of AIS are not specific enough to select the most critical cases. With regard to obstetrical management PPROM infants and infants without PPROM had no differences in rates of spontaneous delivery (12.4 vs. 11.0%), elective C/S (79.3 vs. 79.6%) and emergency caesarean section (8.2 vs. 9.3%). But it has to be considered in future studies that remarkable differences between study centers exist, e.g. the spontaneous delivery rate in PPROM patients differed from 0–47%. In addition to that, the study centers themselves are important covariates in determining neonatal outcomes, i.e. risk for EOS, BPD and PVL.

Besides center-specific variation there might be selection bias of centers with favourable outcomes in GNN which is a well-known limitation of population-based studies [29]. In addition to that, centralization of perinatal services is associated with improved outcome, as it has been well demonstrated by the Swedish EXPRESS study group [30, 31]. This is particularly important for the most vulnerable subgroup of infants < 27 weeks whose mortality and morbidities are largely determined by gestational age at birth [4, 32]. Both medical and logistic aspects have to be considered for the interdisciplinary discussion on how to manage PPROM. In line with that, strategies for better outcome including individualized care and decision making need to be evaluated in a multicenter randomized trial [4, 33, 34].

## Conclusion

Despite limitations our large-scale epidemiological data provide a basis for the important interdisciplinary discussion on PPROM-related outcome. The diagnosis of PPROM per se is not associated with adverse outcome in VLBW infants < 32 weeks apart from a moderately increased risk for BPD. Randomized controlled trials with primary neonatal outcomes are needed to determine which subgroup of VLBW infants benefit from expectant or intentional management of PPROM.
